# Account of Diseases in an Eastern District of London, from the 20th of May to the 20th of June, 1800

**Published:** 1800-07

**Authors:** 


### STATE OF DISEASES IN LONDON.

*Account of Diseases in an Eastern District of London, from the 20th of May to the 20th of June,* 1800.

| | No. of Cases. |
|---|---|
| ACUTE DISEASES. | |
| Typhus mitior | 7 |
| Sore Throat | 2 |
| Small-Pox | 1 |
| Cough | 9 |
| Dyspnœa | 4 |
| Cough with Dyspnœa | 9 |
| Hæmoptoe | 4 |
| Phthisis Pulmonalis | 4 |
| Gastrodynia | 6 |
| Diarrhœa | 20 |
| Colica | 3 |
| Colica Pictonum | 2 |
| Hæmorrhois | 5 |
| Ascites | 4 |
| Anasarca | 6 |
| Hydrothorax | 2 |
| Cephalalgia | 6 |
| Vertigo | 4 |
| Epilepsia | 3 |
| Paralysis | 2 |
| Hysteria | 6 |
| Dysuria | 3 |
| Chronic Rheumatism | 16 |
| PUERPERAL DISEASES. | |
| Peritonitis | 2 |
| Menorrhagia lochialis | 5 |
| INFANTILE DISEASES. | |
| Ophthalmia purulenta | 2 |
| Aphthæ | 5 |
| Rachitis | 1 |
| Scrophula | 4 |
| Hooping Cough | 3 |

Having arrived at that season of the year in which those diseases, which form a large proportion of the list in the winter months, usually decrease in number, or abate in the violence of their symptoms, we might expect that this class of maladies should hardly make their appearance on the list.

The great vicissitude, however, in the temperature of the atmosphere, has protracted some of these complaints to an unusual period. Disorders of the bowels also, which were taken notice of in the last report, form an important part of the present list.

Cases of diarrhœa have been uncommonly numerous; have been protracted to a considerable length, and have been liable to return upon the least irregularity of regimen or diet.

Diseases

